# Trends in the psychosocial and mental health of HIV‐positive women in China from 2015 to 2020: Results from two cross‐sectional surveys

**DOI:** 10.1111/hex.13498

**Published:** 2022-06-20

**Authors:** Qian Wang, Vivian W. I. Fong, Qinghua Qin, Hui Yao, Jiarui Zheng, Xiaoyan Wang, Ailing Wang, Qun Gao, Phoenix K. H. Mo

**Affiliations:** ^1^ Antenatal Health Care Department National Center for Women and Children's Health, China CDC Beijing China; ^2^ School of Public Health and Primary Care The Chinese University of Hong Kong Sha Tin Hong Kong; ^3^ Antenatal Health Care Department Maternal and Child Health Hospital of Guangxi, Zhuang Autonomous Region Nanning China; ^4^ Antenatal Health Care Department Yunnan Maternal and Child Health Care Hospital Kunming China; ^5^ The Chinese University of Hong Kong Shenzhen Research Institute Shenzhen China

**Keywords:** cross‐sectional, HIV, mental health, psychosocial, women

## Abstract

**Background:**

The human immunodeficiency virus (HIV) continues to be one of the major public health challenges in the world. Despite the advancement in medication and changes in views towards HIV in Chinese society, little is known about the changes in the psychosocial and mental health of HIV‐positive women in recent years.

**Objectives:**

The present study examined the change in depression, anxiety, stigma, relationship with the child, intimacy with a partner, and social support from family, friends, and health professionals, for HIV‐positive women in China from 2015 to 2020.

**Methods:**

Two cross‐sectional surveys were conducted in 2015 and 2020, and 429 and 382 HIV‐positive women were recruited from the Women's Health Department in Yunnan and Guangxi, China between November 2015 to May 2016, and November 2019 to January 2020, respectively.

**Results:**

After controlling for significant sociodemographic variables, participants recruited in 2019–2020 had significantly lower levels of depression and anxiety and higher scores on emotional and tangible support from friends. On the other hand, they had lower scores in intimacy with partners and emotional and tangible support from family. No significant changes were found in stigma, relationship with the child, and support from health professionals.

**Conclusion:**

Results provide important information on the changes in psychosocial and mental health, which offer insights into the design of interventions to promote psychosocial and mental health among HIV‐positive women in China.

**Patient or Public Contribution:**

HIV‐positive women contributed to the data of this study. Health care professionals were involved in the discussion of the methods and results.

## 1 INTRODUCTION

The epidemic of human immunodeficiency virus (HIV) continues to be one of the major public health challenges in the world. As of 2020, there are approximately 37.7 million people living with HIV (PLWHIV) worldwide.^1^ Of which, about 20.1 million are women, which accounts for 55% of all adults living with HIV.^2^ In China, despite the overall prevalence of HIV remaining at a low level, the number of new infections and acquired immunodeficiency syndrome (AIDS)‐related deaths continues to rise each year.^2^ From 2015 to 2019, the number of PLWHIV in China increased substantially from 517,423 to 960,000. Among these, 1 in every 3.7 PLWHIV is a woman.^3^ There is ample evidence indicating that not only women are more susceptible to HIV infection than men,^4^ being a woman with HIV‐positive status can mean greater barriers along the HIV treatment continuum due to social norms and gender roles.^5^, ^6^, ^7^

Mental health problems are highly prevalent among PLWHIV and have been widely documented in the literature.^6^, ^8^, ^9^, ^10^ Previous findings have revealed that PLWHIV is more likely to report depression, anxiety, posttraumatic stress disorder, and are at higher risk of violence and substance use than HIV‐negative counterparts.^8^ More specifically, women are found to be more prone to experience poor mental health than their male counterparts after HIV diagnosis.^11^, ^12^, ^13^, ^14^ In the face of their infection, HIV‐positive women often report having depression, shame, self‐blame, feelings of rejection and insomnia.^15^ PLWHIV are also found to have low self‐esteem and suicidal ideations after knowing about their infection.^2^ Poor mental health is associated with various negative outcomes such as poor adherence to treatment regimens, limited social support, poor physical health, risk behaviours such as substance use and lower quality of life,^4^, ^6^, ^14^, ^16^, ^17^ which may result in an elevated risk for all‐cause and AIDS‐related mortality and morbidity, and exert pressure on the health care system.^4^ In addition, due to gender inequality and gender norms embedded in Chinese culture, Chinese women are often constrained by their gender roles and obligations that one needs to fulfil to fit into society. ‘Womanhood’ in China is traditionally perceived as the obligation to have children, be a good mother, and care and self‐sacrifice for the family.^18^ As HIV infection is perceived as defying acceptable social norms and expected roles for women, HIV‐positive Chinese women are judged heavily for not complying with those concepts.^18^ Such family‐centred culture‐induced HIV‐related stigma and discrimination have placed HIV‐positive Chinese women at increased risk of mental health problems.^12^, ^19^ Indeed, HIV care and prevention would not be as effective if psychological issues were not fully addressed.^20^ However, from our understanding, there is only one review that has captured the mental health of PLWHIV from 1998 to 2014 in China,^8^ and little is known about how the mental health of Chinese HIV‐positive women has changed over the years since then. New data is critical to evaluate and inform policies and services that meet the needs of Chinese HIV‐positive women in the future.

Social support refers to one's perception of various supports received from others (e.g., spouse, family, friends or health care professionals). It is generally categorized into two types of support, that is, instrumental support (help or assistance with tangible needs) and emotional support (beliefs of loving and caring, sympathy and understanding). Although support from each source has been found to be associated with various positive outcomes, it is notable that familial support is the primary source of support for PLWHIV in China^21^ and predicts more positive outcomes than support from friends and health care professionals.^22^ However, a number of studies have stated that interpersonal resources may be less available to PLWHIV and such limited resources may be attributable to the HIV‐related stigma and discrimination that prevail in China.^23^, ^24^ According to the China stigma index report in 2009,^2^ 34% of households with HIV‐infected family members were not willing to take care of their HIV‐infected family members or allow them in their household. In addition, a substantial proportion of medical staff (26%), government officials (35%) and teachers (36%) have shown a discriminatory attitude after being told about a person's HIV‐positive status, which can result in inadequate support and suboptimal treatment provided to Chinese HIV‐positive women.

Research has shown that social support is a protective factor that mitigates the negative impact of HIV‐related stigma,^25^, ^26^, ^27^, ^28^ and is associated with better self‐esteem, positive affect, life satisfaction, subjective and health‐related quality of life, and decreased levels of depressive symptomology.^17^, ^22^, ^27^, ^29^ Other positive outcomes, such as increased utilization of HIV‐related preventive and therapy services,^30^ antiretroviral therapy (ART) adherence^7^ and adaptive coping strategies^31^ were also found in HIV‐positive women who perceived stronger social support. Given the beneficial effect of social support in helping PLWHIV to maintain a good mental state and enhance the effect of treatment, it is thus important to understand the current situation of the social support received among HIV‐positive Chinese women and how its utility has changed throughout the years. This new knowledge can provide important insights into the design of future effective interventions that promote social support and mental health of HIV‐positive women in China.

The objective of the present study was to understand the change in psychosocial health, that is, depression, anxiety, stigma, relationship with child, intimacy with partner, and social support from family, friends and health professionals, of HIV‐positive women in China from 2015 to 2020. With the increasing attention to promoting mental health and reducing discrimination and stigma towards PLWHIV in China, it is conjectured that an improvement in psychosocial health will be observed among HIV‐positive women.

## 2 METHODS

### 2.1 Study design

The present study was an analysis of two cross‐sectional surveys among HIV‐positive women recruited in 2015 and 2020. Inclusion criteria were: (1) Chinese women, (2) aged ≥18 years and (3) received a confirmatory HIV+ diagnosis. Exclusion criteria were: (1) have HIV‐positive child(ren), (2) currently pregnant and (3) diagnosed with a psychiatric condition. These women were excluded as their profile of psychosocial and mental health, and source of support received might be different from the general HIV+ women.

### 2.2 Procedure

Participants were recruited from eight Women's Health Departments in three cities located in Yunnan and Guangxi Province, China. These cities are the key pilot areas that have carried out the prevention of mother‐to‐child transmission of AIDS since 2003. They have solid experiences in AIDS prevention and control and women's health. The Women's Health Department provides comprehensive health care services related to women's reproductive health. Its Antenatal Care (ANC) clinic offers universal HIV screening to all pregnant women attending the clinic as part of routine obstetric tests. Those who have screened HIV‐positive during pregnancy receive HIV care services and continue to receive regular follow‐up services at the clinic after they give birth. The two samples of HIV‐positive women were recruited from the ANC between November 2015 to May 2016, and from November 2019 to January 2020.

All HIV‐positive women attending the follow‐up services at the eight ANC clinics during the study period were first screened by the clinician and those who were eligible were invited to participate in the study. Those who agreed to take part were later referred to an interviewer. The study purposes were introduced, participants were assured that refusals would not affect their right to use any services and they could withdraw at any time without being questioned. Informed consent was obtained before the study, and a survey was administered by an interviewer. Participants received a nonmonetary incentive which was worth RMB20 (∼USD2.5) to compensate for the time they spent on the study. Ethics approval was obtained from the institutional Ethics Committee.

### 2.3 Measures

Depression was measured by the 20‐item Center for Epidemiologic Studies Depression Scale (CES‐D).^32^ It has been validated and used in Chinese.^33^, ^34^ Participants rated how often they have experienced depressive symptomatology in the past 7 days, on a 4‐point Likert scale from 0 (rarely or none of the time [less than 1 day]) to 3 (almost or all of the time [5–7 days]). with higher scores reflecting greater severity. Scores of 16, 21 and 25 represent cutoff points for the presence of probable mild, moderate and severe depression, respectively. The Cronbach's *α* was .93 (Sample 1) and .91 (Sample 2)

Anxiety was measured by the 7‐item General Anxiety Disorder scale.^35^ It has been validated and used in Chinese.^36^, ^37^ Items are rated on a 4‐point Likert scale from 0 (never) to 3 (often [almost every day]). A score of 5, 10 and 15 represent cutoff points for the presence of probable mild, moderate and severe anxiety, respectively. The Cronbach's *α* was .96 (Sample 1) and .95 (Sample 2)

Stigma was measured by the 9‐item Self Stigma scale, which was developed to measure self‐stigma among concealable minorities and validated in Chinese community samples.^38^ Items are rated on a 4‐point Likert Scale from 1 (strongly disagree) to 4 (strongly agree), with a higher score indicating a higher level of stigma. The Cronbach's *α* was .95 (Sample 1) and .88 (Sample 2)

Relationship with child was measured by the 15‐item Child–Parent Relationship Scale‐Short Form.^39^ Items assess their perceived relational conflict and closeness with their child on a 5‐point Likert Scale from 1 (definitely does not apply) to 5 (definitely applies), with a higher score indicating a better relationship with the child. The Cronbach's *α* was .71 (Sample 1) and .78 (Sample 2).

Intimacy with partner. Among those who reported having a partner, their level of intimacy with partner was measured by four items that measure the level of closeness, love, conflicts and blame: ‘How close are you and your partner?’, ‘How much do you love each other?’, ‘How often do you and your partner have conflict?’ and ‘How often do you and your partner blame each other?’ The first two items are rated on an 11‐point Likert Scale ranging from 0 (not at all) to 10 (very much), and the last two items are rated on an 11‐point Likert Scale ranging from 0 (always) to 10 (never). A higher score reflects a higher level of intimacy with partner. The Cronbach's *α* was .85 (Sample 1) and .94 (Sample 2)

Social support from family and friends. Two items were used to measure the extent of emotional and tangible (e.g., financial) support from family^40^: ‘How much support did you receive from your family when you needed to talk with someone or needed emotional support?’ and ‘How much support did you receive from your family when you needed tangible support (e.g., financial support)?’ Items are rated on an 11‐point Likert Scale ranging from 0 (none) to 10 (tremendous), with a higher score reflecting a higher level of social support. The same items were also modified to assess emotional and tangible support obtained from friends. The Cronbach's *α* was .90 (Sample 1) and .91 (Sample 2) for social support from friends, and .94 (Sample 1) and .87 (Sample 2) for social support from family.

Support from health professionals. Support from health professionals was measured by the 10‐item consultation and relational empathy measure.^41^, ^42^ Items are rated on a 5‐point Likert Scale from 1 (poor) to 5 (excellent), with a higher score indicating a higher level of support. It has been used in Chinese.^43^ The Cronbach's *α* was .97 (Sample 1) and .98 (Sample 2).

### 2.4 Sample size calculation

A previous study has shown that the mean score of depression among HIV‐positive individuals in China, as measured by the CES‐D, was 24 (SD = 6.5).^44^ A sample size of 390 per group would allow us to detect a difference between groups with a small effect size (*d* = 0.2), power = 0.8, *α *= .05, two‐sided test (GPower).

### 2.5 Analysis

Descriptive statistics on socio‐demographic information of participants from both samples were first presented. Differences in sociodemographic variables were compared using an independent sample *t*‐test (for continuous variables) or *χ* ^2^ test (for categorical variables). To compare the trends in psychosocial and mental health outcomes, the differences in the outcomes between two samples were first tested using an independent sample *t*‐test. Analysis of covariance tests were also conducted, controlling for sociodemographic variables that were statistically significant between the two samples at the *p* < .05 level. The pairwise deletion was used to deal with missing data. All analyses were conducted using SPSS Statistics 26.

## 3 RESULTS

### 3.1 Sociodemographic characteristics of participants

A total of 429 (Sample 1) and 382 (Sample 2) participants were recruited from the two samples, respectively. The mean age of the participants was 39.4 years (Sample 1) and 37.97 years (Sample 2): 44.2% (Sample 1) and 63.5% (Sample 2) had received a secondary level of education or above; and 81.3% and 76.9% of Samples 1 and 2, respectively, were married. They had been diagnosed with HIV for 7.65 (Sample 1) and 8.85 years (Sample 2); 40.0% (Sample 1) and 76.4% (Sample 1) were in an asymptomatic stage. The majority (90.2% in Sample 1 and 98.1% in Sample 2) were on antiretroviral treatment. Results from independent *t*‐tests and *χ* ^2^ tests revealed a significant difference in age, education level, marital status, duration of HIV diagnosis, disease stage and status of ART between the samples, with participants from Sample 2 being younger, more educated, less likely to be married, having a shorter duration of HIV diagnosis, and more likely to be in the asymptomatic stage and on ART (Table 1).

**Table 1:** Sociodemographic characteristics of participants

	Sample 1 (2015)	Sample 2 (2020)	Differences between groups
Age	*M* = 39.40 years, SD = 10.57	*M* = 37.96 years, SD = 7.49	*t*(797) = 2.25, *p* < .001^a^
Education level			*χ* ^2^(4) = 71.85, *p* < .001^b^
Illiterate	93 (21.7%)	10 (2.6%)	
Primary	146 (34.0%)	129 (33.9%)	
Junior secondary	161 (37.5%)	204 (53.5%)	
Senior secondary	19 (4.4%)	27 (7.1%)	
College or above	10 (2.3%)	11 (2.9%)	
Marital status			*χ* ^2^(4) = 36.57, *p* < .001^b^
Single	9 (2.1%)	10 (2.6%)	
Married	348 (81.3%)	293 (76.9%)	
Cohabitation	19 (4.4%)	6 (1.6%)	
Divorced	6 (1.4%)	40 (10.5%)	
Widowed	46 (10.7%)	32 (8.4%)	
Financial status	*M* = 2.68, SD = 0.76	*M* = 2.76, SD = 0.67	N.S.^a^
Duration of HIV diagnosis	*M* = 7.65 years, SD = 3.70	*M* = 8.85 years, SD = 4.30	*t*(801) = −4.23, *p* < .001^a^
Disease stage			*χ* ^2^(3) = 113.96, *p* < .001^b^
Asymptomatic	170 (40.0%)	292 (76.4%)	
Symptomatic	17 (4.0%)	13 (3.4%)	
AIDS	177 (41.6%)	62 (16.2%)	
Not sure	61 (14.4%)	15 (3.9%)	
On antiretroviral treatment	370 (90.2%)	363 (98.1%)	*χ* ^2^(1) = 21.42, *p* < .001^b^
Number of children	*M* = 1.88, SD = 0.94	*M* = 1.85, SD = 0.95	N.S.^a^

*Note*: The sample size for each variable varied slightly due to missing data: age (*n* = 429 for Sample 1, 381 for Sample 2), education level (*n* = 429 for Sample 1, 381 for Sample 2), marital status (*n* = 428 for Sample 1, 381 for Sample 2), financial status (*n* = 425 for Sample 1, 381 for Sample 2), duration of HIV diagnosis (*n* = 429 for Sample 1, 381 for Sample 2), disease stage (*n* = 425 for Sample 1, 382 for Sample 2), on antiretroviral treatment (*n* = 410 for Sample 1, 370 for Sample 2), number of children (*n* = 410 for Sample 1, 381 for Sample 2).

Abbreviations: AIDS, acquired immunodeficiency syndrome; HIV, human immunodeficiency virus; *M*, mean.

^a^ Results from independent sample *t*‐test.

^b^ Results from *χ* ^2^ test.

### 3.2 Trends in psychosocial and mental health

Results from independent samples *t*‐tests showed that participants from Sample 2 had significantly less depression and anxiety, had more intimacy with partner, and received more emotional and tangible support from friends than participants from Sample 1. On the other hand, they received less emotional and tangible support from family compared to those from Sample 1. These differences remained significant after adjusting for significant sociodemographic variables. No significant differences were found in stigma, relationship with child and support from health professionals (Table 2).

**Table 2:** Trends in the psychosocial and mental health of HIV‐positive women from 2015 to 2020

	Sample 1 (2015)	Sample 2 (2020)	Differences between groups^a^	Differences between groups after controlling for significant sociodemographic variables^b^
Depression	*M* = 20.78, SD = 11.26	*M* = 17.32, SD = 10.35	*t*(805) = 4.53, *p* < .001	*F*(1, 746) = 6.52, *p* < .01
Anxiety	*M* = 12.80, SD = 5.14	*M* = 11.63, SD = 4.81	*t*(805) = 3.31, *p* < .001	*F*(1, 746) = 3.15, *p* < .05
Stigma	*M* = 2.80, SD = 0.45	*M* = 2.79, SD = 0.52	N.S.	N.S.
Relationship with child	*M* = 3.03, SD = 0.34	*M* = 3.04, SD = 0.46	N.S.	N.S.
Intimacy with partner	*M* = 8.32, SD = 1.99	*M* = 7.38, SD = 2.71	*t*(676) = 5.16, *p* < .000	*F*(1, 630) = 34.62, *p* < .05
Emotional support from family	*M* = 7.86, SD = 2.85	*M* = 7.23, SD = 2.78	*t*(802) = 3.16, *p* < .01	*F*(1, 744) = 9.99, *p* < .05
Tangible support from family	*M* = 7.55, SD = 3.07	*M* = 7.09, SD = 2.80	*t*(802) = 2.19, *p* < .05	*F*(1, 744) = 6.80, *p* < .01
Emotional support from friends	*M* = 4.89, SD = 3.82	*M* = 6.01, SD = 2.97	*t*(801) = −4.61, *p* < .001	*F*(1, 743) = 10.33, *p* < .05
Tangible support from friends	*M* = 4.35, SD = 3.78	*M* = 5.41, SD = 3.08	*t*(800) = −4.33, *p* < .001	*F*(1, 742) = 8.35, *p* < .05
Support from health professionals	*M* = 3.40, SD = 0.84	*M* = 3.47, SD = 0.86	N.S.	N.S.

Abbreviations: ANCOVA, analysis of covariance; HIV, human immunodeficiency virus; *M*, mean.

^a^ Results from independent sample *t*‐test.

^b^ Results from ANCOVA test adjusting for significant sociodemographic variables: age, education level, marital status, duration of HIV diagnosis, disease stage, on antiretroviral treatment.

## 4 DISCUSSION

With advanced and increased access to ART, the life expectancy of HIV‐positive women has substantially improved. While HIV is no longer a terminal condition, mental health problems are often comorbid with HIV infection and associated with poorer adherence to treatment regimens, lower quality of life, increased substance use and involvement in casual relationships, these consequences are critical to HIV care and prevention.^4^, ^8^, ^17^ However, limited existing research has captured and provided insights into the ever‐changing situation of the mental health of HIV‐positive women in China over time. The present study has provided important insights that the mental health of HIV‐positive Chinese women has improved from 2015 to 2020. However, although both levels of depression and anxiety have reduced significantly throughout the years, it is important to note that both indicators of mental health in this study still remained at moderate levels, highlighting the continuing need for mental health services that address and ameliorate the mental health problems experienced by this population. Moreover, it remains unclear what has contributed to the improvements in the mental health of HIV‐positive women in China over the 5 years. Future research is warranted in this regard, which could facilitate the development of future policies and interventions to promote further improvement of mental health in this population. For instance, the United States has provided the roadmap to end the HIV pandemic within a decade, with a vision to empower PLWHIV by engaging them in community programme design and implementation.^45^

Consistent with previous literature,^21^ our findings revealed that HIV‐positive Chinese women reported having received the strongest tangible and emotional support from family than the other two sources, that is, support from friends and health professionals. However, given the findings of the improvement in the mental health of Chinese HIV‐positive women and the positive relationship between familial support and mental health, it is surprising to note that familial support, both tangible and emotional, and intimacy with partners have reduced significantly from 2015 to 2020. The family has been evidenced as one of the most significant sources of stress for PLWHA. While increasing efforts have been placed in promoting HIV disclosure to family members and encouraging family involvement in HIV care, the greater involvement of family members might further exacerbate stress and conflict.^46^, ^47^ There is an urgent need to promote familial support in this population, as stated in the existing literature, HIV‐positive women, in general, are in great need of material and psychological support from their families and that support from the family will lead to multiple positive impacts for PLWHIV, including mental health.^11^, ^14^ In a collectivist culture, HIV‐positive Chinese women would benefit greatly from a family‐centred intervention. Literature has documented early evidence for the effect of family‐centred approaches on increasing communication between HIV‐positive women and their spouses, their uptake of HIV testing, and adherence to ART in several collectivist countries, such as Kenya and Uganda.^48^ Moreover, examining the underlying causes of the lowering of familial support is also critical and warranted in future research.

Notably, the present study has found that both tangible and emotional support from friends have increased significantly from 2015 to 2020. Friends were often perceived as more supportive than family members.^22^, ^49^ Peer support allows individuals to receive emotional comfort without burdening others with problems or stress, it can lessen the fear of repercussion on support systems^23^ and therefore, is especially protective for mental health.^50^, ^51^ A possible explanation for the increase in support from friends may be attributed to the advancement in digital technologies and the popularization of social media in recent years, where online social networks have revolutionized the way friends connect. In an anonymous and easily accessible environment, online social groups on social media, e.g. Weibo, offer PLWHIV opportunities to share personal experiences and obtain various support without being stigmatized.^52^ They can be important platforms for HIV‐positive women to obtain support and information in a convenient manner.

Crucially, it is also important to note that the levels of HIV‐related stigma reported in our findings have not changed from 2015 to 2020. In the last decade, the Chinese government has made substantial efforts in response to the HIV epidemics in China by implementing major HIV prevention and control regulations, that is, The Four Frees and One Care programme offers free services to PLWHIV, including ART, counselling and testing and prevention of mother‐to‐child transmission. However, it is not until recently that the Chinese government has started to mention the negative impact of HIV‐related stigma and propose various health education campaigns to the public. Future research is needed to understand and monitor the extent to how which changes in HIV‐related policy and responses influence the levels of stigma and other mental health outcomes of HIV‐positive individuals in the future.

The present study has some limitations. First, the findings of the present study may not be representative of all HIV‐positive women in China as the participants were recruited from the ANC. Second, one should be cautious when interpreting the results as self‐reported data may have introduced reporting bias due to social desirability. Third, social support was measured by a single self‐constructed item. As stated in a previous study, fathers and brothers were found to be relatively less supportive than other family and friends.^49^ A single item may not be able to capture each member of the social network and the supportive role they provide, as each support may weigh differently. Lastly, the present study included a convenience sample, and therefore may not be generalizable to the population. Further research is needed to explore the associations between variables in a randomly selected sample in more detail.

Despite these limitations, the findings of the present study provide important preliminary information that can help policymakers, researchers, health educators and clinicians in understanding the social support received and the mental health of HIV‐positive women in China from 2015 to 2020. The study has highlighted the continued need and efforts to reduce HIV stigma and discrimination and to promote social support within this population, especially support from family. It has also provided important insights for the design of interventions for better psychosocial and mental health among HIV‐positive women in China. Future research should continue to monitor the stigma index trend, utilization of social support and mental health of this population and its associated factors. Future actions should focus on developing effective strategies that are tailored to the needs of HIV‐positive women in China and supporting them on a lifelong journey to wellness.

## AUTHOR CONTRIBUTIONS

Qian Wang and Phoenix K. H. Mo contributed to the conceptualization and study design; Qinghua Qin, Hui Yao, Jiarui Zheng, Xiaoyan Wang, Ailing Wang and Qun Gao performed the data collection; Vivian W. I. Fong and Phoenix K. H. Mo finished the data analysis and wrote the original manuscript; all authors have reviewed and edited the manuscript, and agreed to the published version of this manuscript.

## CONFLICTS OF INTEREST

The authors declare no conflicts of interest.

## Data Availability

The data are not publicly available due to ethical considerations as it could compromise the privacy of research participants.
